# Regulation of Ergosterol Biosynthesis in Pathogenic Fungi: Opportunities for Therapeutic Development

**DOI:** 10.3390/microorganisms13040862

**Published:** 2025-04-10

**Authors:** Lingyun Song, Sha Wang, Hang Zou, Xiaokang Yi, Shihan Jia, Rongpeng Li, Jinxing Song

**Affiliations:** 1 The Key Laboratory of Biotechnology for Medicinal and Edible Plant Resources of Jiangsu Province, School of Life Sciences, Jiangsu Normal University, Xuzhou 221116, China; 2020221696@jsnu.edu.cn (L.S.); 3020222540@jsnu.edu.cn (X.Y.); jiashihan@jsnu.edu.cn (S.J.); 2 Huzhou Key Laboratory of Precise Prevention and Control of Major Chronic Diseases, Huzhou University, Huzhou 313000, China; wangsha@zjhu.edu.cn; 3 Antibiotics Research and Re-Evaluation Key Laboratory of Sichuan Province, Chengdu University, Chengdu 610059, China; zouhang1108@cdu.edu.cn; 4 Qian Xuesen Collaborative Research Center of Astrochemistry and Space Life Science, Institute of Drug Discovery Technology, Ningbo 315211, China

**Keywords:** ergosterol biosynthesis, pathogenic fungi, *Aspergillus fumigatus*, antimicrobial resistance, *erg11A/cyp51A*

## Abstract

Ergosterol plays a dual role in fungal pathogenesis and azole resistance, driving key advancements in the understanding of its biosynthesis regulation. This review integrates the latest research progress on the regulation of fungal ergosterol biosynthesis and its role in drug resistance and pathogenicity. We comprehensively discuss the functions of key enzymes (such as Erg11p/Cyp51A, Erg6p, Erg3p, and Erg25p) in the mevalonate, late, and alternative pathways. Notably, we highlight the complex regulation of *cyp51A* expression by factors such as SrbA, AtrR, CBC, HapX, and NCT in *Aspergillus fumigatus*, and elucidate the distinctive roles of Upc2, Adr1, and Rpn4 in *Candida* species. Importantly, we summarize recent discoveries on the CprA-dependent regulation of Cyp51A/Erg11p and heme-mediated stability control. Based on these findings, we propose innovative antifungal strategies, including dual-target inhibition and multi-enzyme inhibition by natural products, which provide novel insights and potential directions for the development of next-generation antifungal therapies.

## 1. Introduction

Pathogenic fungi, including species such as *Aspergillus*, *Candida*, and *Cryptococcus*, represent a growing global health threat, with invasive infections leading to alarmingly high mortality rates, especially among immunocompromised individuals [1,2,3,4,5]. The rise of pan-resistant fungal strains, driven by widespread antifungal use in both agricultural and medical settings, has intensified this crisis, rendering traditional antifungal therapies increasingly ineffective [6,7,8]. This issue is further exacerbated by the evolutionary paradox faced in antifungal drug development: although fungi share many fundamental biological processes with humans due to their eukaryotic nature, this close evolutionary relationship complicates the identification of unique therapeutic targets and heightens the risk of adverse effects on human cells. As a result, current treatment strategies have focused on targeting pathways that are significantly different between fungi and humans, such as cell wall biosynthesis and sterol metabolism, to minimize collateral damage.

Invasive fungal infections are primarily treated with three major classes of antifungal drugs: azoles, echinocandins, and polyenes [9]. Azole drugs work by inhibiting lanosterol 14α-demethylase in the fungal cell membrane, thereby blocking ergosterol synthesis and compromising membrane integrity. Azole resistance primarily results from mutations in the *erg11p* gene that alter the target enzyme’s structure, or from mutations in regulatory genes that lead to the overexpression of drug efflux pumps, reducing intracellular drug concentrations [10]. Polyene drugs (such as amphotericin B) bind directly to ergosterol, disrupting membrane permeability and causing cell death; their resistance is often associated with changes in the ergosterol biosynthetic pathway or alterations in membrane components. Echinocandins (e.g., caspofungin) inhibit β-(1,3)-D-glucan synthase, disrupting cell wall synthesis, but FKS mutations can lower enzyme affinity, leading to resistance [9,11]. The misuse of these antifungal drugs in both medicine and agriculture has significantly accelerated the evolution of resistance. Clinically, the prolonged prophylactic use of broad-spectrum azoles continuously exerts selective pressure, enabling fungi like *A. fumigatus* to develop azole resistance through mutations in the *cyp51A* gene [12]. In agriculture, the extensive use of azole fungicides exposes various *Aspergillus* species in the environment to sublethal doses over extended periods, driving mutations in the *cyp51A* gene; these environmentally resistant strains can then be transmitted to humans via airborne routes, resulting in treatment difficulties [12]. In summary, these misuses collectively have significantly increased both the spread of resistant fungi and the difficulty of treatment, underscoring the urgent need for precise dosing, resistance monitoring, and the development of new drugs targeting novel sites to address this global threat.

Sterols, essential structural and functional components of the plasma membrane, play a crucial role in regulating membrane dynamics, fluidity, and cellular responses to environmental stress [13,14]. Among these, ergosterol—a C28 sterol found exclusively in fungi—has emerged as a central player in fungal survival. Its biosynthesis involves a complex enzymatic pathway that diverges from the cholesterol (C27 sterol) biosynthesis pathway in mammals, making it an ideal pharmacological target with the potential to disrupt fungal viability without affecting human cells [15]. Recent research has expanded our understanding of ergosterol’s role beyond membrane structure, highlighting its function as a molecular rheostat that controls fungal virulence, drug resistance, and immune evasion [16,17]. These findings have reignited interest in ergosterol homeostasis as a promising target for polypharmacological strategies that aim to disrupt multiple aspects of fungal pathogenicity simultaneously.

While previous reviews have examined various facets of antifungal resistance and ergosterol biosynthesis, this article stands out by offering a comprehensive synthesis of the latest regulatory mechanisms involved in ergosterol biosynthesis in fungi. We will highlight recent advancements in understanding the complex signaling pathways and genetic factors that regulate this process—areas that have not received as much attention in the earlier literature. Furthermore, we will explore the implications of these findings for the development of targeted antifungal therapies, providing new insights into potential strategies for overcoming resistance. This updated summary is particularly significant, as it bridges the gap between fundamental research and clinical applications, addressing the urgent need for innovative antifungal approaches in the face of increasing resistance.

## 2. Methodology: Systematic Literature Review Framework

### 2.1. Data Sources and Search Strategy

To ensure a comprehensive and multidisciplinary approach to antifungal research, we collected primary research from the following key databases: ① PubMed, a biomedical database focused on antifungal drug trials and mechanisms; and ② Web of Science Core Collection, a multidisciplinary database covering life sciences, chemistry, and pharmacology. The literature search strategy was executed by optimizing Boolean combinations of the following keywords: pathogenic fungi (e.g., “*Candida*” OR “*Aspergillus*” OR “*Cryptococcus*” OR “*Fusarium*”), the ergosterol biosynthesis pathway (e.g., “ergosterol biosynthesis” OR “sterol regulation” OR “Cyp51” OR “Erg6p” OR “Erg25p”), therapeutic targets (e.g., “azole resistance” OR “antifungal drug development” OR “polypharmacology” OR “nanoparticle delivery”), and mechanisms of action (e.g., “sterol transcriptional regulation” OR “SREBP” OR “efflux pumps” OR “biofilm disruption”). This systematic search strategy has facilitated the identification of the relevant literature covering pathogenic fungi, the ergosterol biosynthesis pathway, antifungal drug resistance mechanisms, and the development of novel therapeutic targets.

### 2.2. Inclusion and Exclusion Criteria

The literature selection was based on a set of rigorous inclusion and exclusion criteria. We included peer-reviewed articles published between January 2000 and December 2025 to capture a 25-year span of post-genomic advancements in fungal sterol biology. Studies focused on human–pathogenic fungi—specifically *Candida albicans*, *A. fumigatus*, and *Cryptococcus neoformans*—were prioritized. The review encompassed research articles, reviews, and clinical trials that addressed the molecular mechanisms of ergosterol biosynthesis and regulation, antifungal drug development targeting sterol pathways, and evolutionary adaptations related to sterol metabolism in pathogenic fungi. Conversely, the literature was excluded if it was published in non-English languages, consisted of non-peer-reviewed content such as conference abstracts or redundant datasets, or lacked mechanistic detail, as in the case of clinical case reports or studies without molecular profiling of sterol pathways.

## 3. Mechanistic Insights and Functional Implications of Ergosterol Biosynthetic Pathway in Pathogenic Fungi

**Figure 1:**
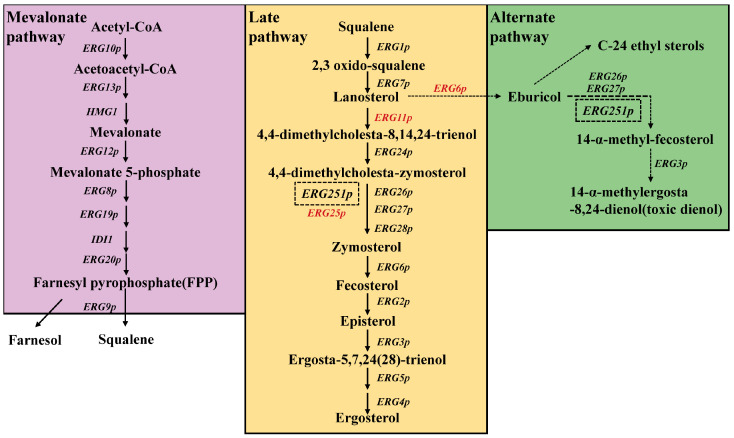
The biosynthesis pathway of ergosterol in fungi. The diagram illustrates the key intermediates, final products, and enzymes involved in ergosterol synthesis. Different colors represent distinct modules, including the mevalonate pathway, late-stage ergosterol pathway, and alternative pathways.

Ergosterol biosynthesis, a critical process for fungal membrane formation, is highly conserved within the Saccharomycotina subphylum, which includes genera such as *Candida* and *Saccharomyces cerevisiae* [18,19]. The biosynthetic pathway is a complex metabolic network involving over 20 enzymes, although species-specific variations in gene duplication and expression patterns exist [20,21,22]. This pathway can be categorized into three major branches: the mevalonate pathway, the late pathway, and the alternative pathway (Figure 1). Together, these pathways ensure the stability and functionality of membranes in pathogenic fungi. The mevalonate pathway generates farnesyl diphosphate (FPP), a key precursor in ergosterol biosynthesis. FPP is then dephosphorylated to produce farnesol, a quorum-sensing molecule that regulates the yeast-to-hypha transition and biofilm formation in *C. albicans* [23]. The enzyme Erg9p catalyzes the condensation of two FPP molecules into squalene, a critical step in steroid biosynthesis. The late pathway subsequently converts squalene into ergosterol through a series of enzymatic reactions. Lanosterol 14α-demethylase (Erg11p), a rate-limiting enzyme in this branch, is a primary target of azole antifungals [9]. Azole inhibition of Erg11p disrupts ergosterol production, causing the accumulation of lanosterol, which is redirected into the alternative pathway. This interruption significantly impacts fungal viability and drug susceptibility. The alternative pathway involves enzymes such as Erg6p and Erg3p, which catalyze key steps in the synthesis of toxic dienols [24]. Modifications in these enzymes contribute to drug resistance in *Candida* species. For example, dysfunction of Erg6p reduces the sensitivity of *Candida glabrata* to nystatin and polyenes [25], while inactivation of Erg3p confers azole resistance in *C. albicans*, *Candida parapsilosis*, and *Candida dubliniensis*, as well as polyene resistance in *C. albicans* and *Candida lusitaniae* [26,27,28,29]. Loss of Erg3p activity reduces toxic dienol production, allowing for the accumulation of 14α-methylfecosterol, which supports fungal survival under azole stress. Notably, Erg3p inactivation can also rescue the lethality seen in Erg11p deletion mutants across species [30]. Both the late and alternative ergosterol biosynthesis pathways utilize C-4 sterol methyl oxidase, a key component of the C-4 demethylation complex that removes two methyl groups from the sterol molecule’s C-4 position. In *S. cerevisiae*, Erg25p is the sole C-4 methyl oxidase required for growth [31,32]. However, in *A. fumigatus* and *C. albicans*, two oxidases, Erg25p and Erg251p, exist, with Erg251p serving as the dominant enzyme in the alternative pathway of *C. albicans* [33]. Dysfunction of Erg251p leads to the accumulation of 14α-methylfecosterol and a reduction in toxic dienol synthesis, enhancing azole tolerance. Paradoxical observations, such as decreased fluconazole (FLC) sensitivity in Erg251p transposon mutants, underscore the influence of genetic background and environmental conditions on phenotypic outcomes [20,34]. Disruptions in Erg251p function in *C. albicans* lead to various pleiotropic effects. Heterozygous deletion confers azole tolerance in diploid strains, with aneuploidy (chromosomes 3/6) further amplifying resistance. Homozygous deletion enhances fungal fitness at low fluconazole concentrations but impairs growth in nutrient-rich environments, particularly at low cell densities. Altered sterol profiles in these mutants reduce toxic dienol accumulation while increasing non-toxic sterol levels under fluconazole stress, leading to dysregulation of transcription, hyphal development, and stress responses. Moreover, homozygous deletion of Erg251p significantly reduces systemic infectivity in murine models, suggesting its role in fungal virulence [33]. Research has shown that the ergosterol biosynthetic pathway in *A. fumigatus* comprises 14 sterol intermediates, with the final products including not only ergosterol but also a unique secondary metabolite—C-24 ethyl sterol [35]. Notably, the fungus catalyzes this branch reaction via a C-24 sterol methyltransferase encoded by the *erg6p* gene, and this process exhibits significant transcriptional repression under azole antifungal drug stress. This suggests that *A. fumigatus* may dynamically regulate the C-24 ethyl sterol synthesis pathway in response to drug pressure [35]. Current research indicates that C-24 ethyl sterol, a characteristic sterol of plants (since mammals cannot synthesize it due to their lack of C-24 alkylation capability), is extremely rare in fungi. Although this compound has been detected in *A. fumigatus*, its biological function remains doubly unknown—both its role in basic fungal metabolism and its molecular mechanisms in pathogenesis have not been clarified. This cross-kingdom sterol biosynthetic capability may provide important clues for understanding the environmental adaptation strategies of *A. fumigatus* and for developing novel antifungal targets. While key enzymes such as Erg11p, Erg251p, Erg3p, and Erg6p have been extensively studied, the physiological roles of many components in the ergosterol biosynthetic pathway remain unclear. Several critical knowledge gaps persist: ① Species-specific enzyme diversification: The molecular basis of functional divergence among ergosterol-related enzymes across different fungal pathogens remains to be elucidated. ② Metabolic plasticity: The interplay between sterol reprogramming, phenotypic adaptation, and environmental stress responses requires further exploration. ③ Dynamic regulation: The mechanisms by which fungal pathogens modulate this pathway in response to host-imposed stresses, such as immune attacks and nutrient limitations, remain poorly understood. In summary, ergosterol biosynthesis is central to fungal membrane dynamics, stress adaptation, drug resistance, and pathogenicity. A deeper understanding of the regulatory networks, enzyme interactions, and evolutionary adaptations in this pathway will provide valuable insights for developing novel antifungal strategies.

## 4. Regulation of Ergosterol Biosynthesis in Pathogenic Fungi

### 4.1. Transcriptional Regulation of Ergosterol Biosynthesis

The transcriptional regulation of ergosterol biosynthesis in pathogenic fungi is intricately controlled by a network of regulatory factors that govern the expression of key ergosterol biosynthesis genes. Recent breakthroughs have dramatically expanded our understanding of these regulatory mechanisms, shedding light on previously unexplored aspects of transcriptional control, particularly in major fungal pathogens. This section highlights the novel transcriptional regulators identified in recent studies, along with the newly discovered pathways and interactions that fine-tune ergosterol biosynthesis. By focusing on the latest findings, we emphasize how these insights are reshaping our approach to targeting ergosterol biosynthesis in the fight against fungal infections.

#### 4.1.1. Transcriptional Network in *A. fumigatus*

**Figure 2:**
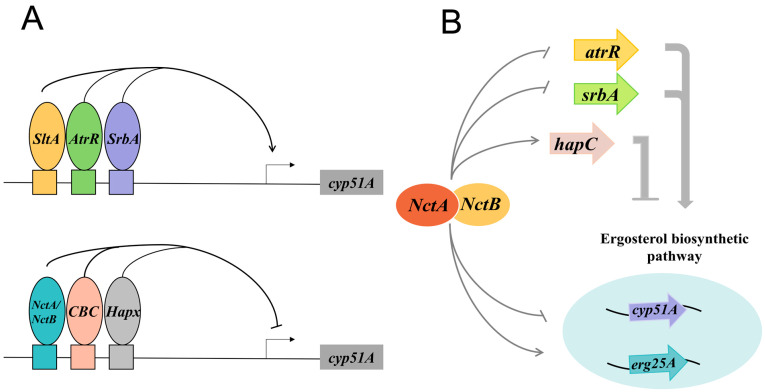
Transcription factors involved in ergosterol biosynthesis in *A. fumigatus*. (**A**) The transcription factors SrbA, AtrR, and SltA function as positive regulators, promoting the expression of *cyp51A*. In contrast, the transcription factors NctA/NctB, the CCAAT-binding complex (CBC), and HapX serve as negative regulators, inhibiting *cyp51A* expression. (**B**) The NCT complex suppresses the expression of *srbA*, *atrR*, and *erg11A* while simultaneously activating *hapC*.

Recent breakthroughs have unveiled a sophisticated transcriptional network regulating ergosterol biosynthesis in the pathogenic fungus *A. fumigatus*, offering new insights into antifungal resistance mechanisms [36,37,38,39,40,41,42]. Central to this network is the 34 bp promoter region of *erg11A/cyp51A*, which integrates signals from multiple transcription factors (TFs) through dynamic interactions (Figure 2).

##### Core Regulatory Axis: SrbA-AtrR Synergy

Building on conserved sterol-regulatory mechanisms observed in *Schizosaccharomyces pombe*, where Sre1 controls *erg3p* and *erg11p* expression [43], *A. fumigatus* employs its SREBP homolog, SrbA, to regulate ergosterol biosynthesis. SrbA binds to the sterol response element (SRE) within the *erg11A/cyp51A* promoter, directly activating *erg3*, *erg24A*, and *erg25A*, alongside the core gene *erg11A/cyp51A* [42,44,45]. Recently, another transcription factor, AtrR, has been identified as a key positive regulator of *erg11A/cyp51A* in *A. fumigatus*. AtrR, a zinc finger transcription factor, binds to a second site within the 34 bp promoter region of *erg11A/cyp51A*, known as the AtrR response element (ATRE), thereby enhancing *erg11A/cyp51A* expression [39]. Chromatin immunoprecipitation sequencing (ChIP-seq) data indicate that the AtrR binding motif (CGG-Nx-CCG) is located between positions 314 and 292 of the *erg11A/cyp51A* promoter, adjacent to the SrbA binding site [39]. This spatial proximity suggests a cooperative regulatory mechanism in which AtrR and SrbA function together to modulate *erg11A/cyp51A* transcription.

##### Dynamic Repressive Networks

In addition to the positive regulators, *erg11A/cyp51A* expression is also modulated by two negative transcriptional regulators which act through interconnected repressive systems to fine-tune ergosterol biosynthesis in response to environmental cues. ① The CBC-HapX Axis: The CCAAT-binding complex (CBC), a heterotrimer consisting of HapB, HapC, and HapE, along with the iron-responsive transcription factor HapX, both function to suppress *erg11A/cyp51A* expression [38,41,44,46,47]. The CBC binds to the 34 bp region of the *erg11A/cyp51A* promoter, while HapX binds downstream of this region [48,49]. The CBC complex competes with SrbA for binding to the CGAAT motif within the *erg11A/cyp51A* promoter (Figure 2). This competition establishes a dynamic equilibrium: CBC deletion enhances SrbA binding capacity, leading to upregulated *erg11A/cyp51A* expression and consequently increased azole resistance [38,41]. Recent mechanistic insights reveal that the CBC’s role extends beyond steric hindrance; it regulates SrbA at multiple levels, including modulation of *srbA* mRNA stability, control of proteolytic cleavage of the SrbA precursor, and inhibition of mature SrbA’s nuclear translocation [41]. ② NCT-Mediated Cascade emerging evidence identifies the negative cofactor 2 (NCT) complex (composed of NctA and NctB) as a master regulator within this network (Figure 2). NCT exerts multilevel control by directly repressing *srbA*, *atrR*, and *erg11A* while activating *hapC* to amplify CBC-mediated repression [36]. This dual regulatory mode explains why inactivation of NCT leads to ergosterol hyperaccumulation, suggesting its role as a central metabolic gatekeeper. Collectively, these repressive mechanisms converge on the 34 bp promoter hub (Figure 2), forming a responsive system that balances sterol synthesis with cellular stress adaptation. The discovery of NCT-mediated hierarchical control and the CBC’s non-canonical regulation of SrbA maturation offers unprecedented insights into the evolution of fungal azole resistance.

##### Beyond the 34 bp Hotspot: SltA as a Novel Master Regulator

While canonical regulators converge on the 34 bp *erg11A/cyp51A* promoter hub, a paradigm-shifting discovery reveals that the fungal-specific C2H2 zinc finger protein SltA operates through a distinct regulatory axis (Figure 2). Unlike SrbA or AtrR, SltA bypasses the conserved 34 bp region and instead binds to evolutionarily divergent 5′-AGGCA-3′ motifs within the promoters of *erg11A/cyp51A*, *erg13A*, and *erg24A* [37]. This DNA-binding mechanism critically depends on conserved cysteine/histidine residues within SltA’s DNA-binding domain, as mutagenesis of these sites abolishes both DNA binding and azole tolerance. Functionally, SltA deletion not only hypersensitizes wild-type strains to azoles but also overrides resistance in clinical isolates carrying *cyp51A* mutations (e.g., TR34/L98H). This phenotype is conserved across genetic backgrounds, indicating that SltA functions independently of classical resistance pathways. Targeting its DNA-binding interface could ① resensitize resistant strains by collapsing ergosterol synthesis, ② synergize with existing azoles through an orthogonal mechanism, and ③ reduce resistance evolution due to SltA’s dominance in sterol pathway regulation [37,46]. This “network override” strategy—exploiting master regulators outside core resistance mechanisms—represents a conceptual leap in antifungal development, offering a novel approach to overcoming azole resistance.

#### 4.1.2. Spatial Hierarchy of Transcription Factor Binding Sites in erg11A/cyp51A

##### Regulation

The transcription factors SrbA, AtrR, CBC, and HapX play crucial roles in regulating *erg11A/cyp51A* expression, yet the spatial hierarchy and functional contributions of their binding sites remain incompletely understood. Although azole-induced upregulation of *erg11A/cyp51A* is well documented, the precise mechanistic basis by which these transcription factors coordinate drug-responsive expression remains unclear. This uncertainty extends to the poorly characterized transcriptional amplification mediated by the 34 bp tandem repeat (TR34) in the *erg11A/cyp51A* promoter [50,51]. Emerging evidence from systematic deletion analyses now clarifies distinct regulatory roles: targeted disruption of either the SrbA-binding SRE or AtrR-binding ATRE elements in the wild-type promoter reduces basal *erg11A/cyp51A* expression, correlating with hypersusceptibility to azoles [42], whereas ablation of the HapX-responsive HXRE element paradoxically elevates gene expression and confers azole resistance [42]. Together, these findings establish a dual-control paradigm in which SRE and ATRE act as transcriptional enhancers essential for baseline expression, while HXRE functions as a repressive element that constrains overexpression.

**Figure 3:**
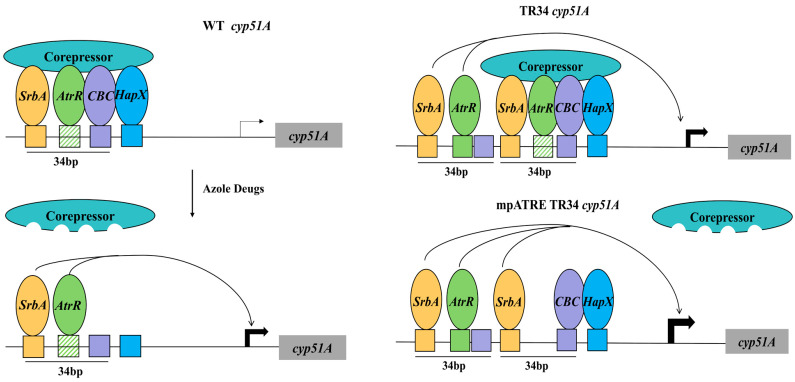
A diagram of potential roles of trans- and cis-acting factors at wild-type and TR34 *cyp51A* promoters. A hypothetical corepressor is pictured that makes multivalent contacts with the key regulators of *cyp51A* transcription. The proximal ATRE is indicated by a green hatched box. Other binding sites are color-coded with their respective regulators. Azole drugs trigger corepressor dissociation and gene activation. In the case of the TR34 promoter (right-hand diagrams), the distal SRE and ATRE in the upstream 34 bp repeat can bypass corepressor function and activate transcription. The 34 bp tandem repeats do not include the HXRE but maintain a CBC binding site. Interaction of the CBC with the adjacent HXRE is required for strong binding of these factors. Exposure of the TR34 *cyp51A* gene to azole drugs or loss of the pSRE or pATRE (shown here) triggers strong induction of expression. Adapted from Figure 2C in reference [42].

The TR34 promoter adds another layer of complexity. It contains duplicated SRE and ATRE sites, along with CBC binding sites, but does not duplicate HXRE. To better define these sites, they are categorized as proximal (pSRE/pATRE) or distal (dSRE/dATRE) based on their relative position to the transcription start site. Intriguingly, in the TR34 promoter, deletion of pSRE or pATRE paradoxically increases *erg11A/cyp51A* expression and reduces azole sensitivity, suggesting a negative regulatory role distinct from their function in the wild-type promoter. Conversely, deletion of dSRE or dATRE results in decreased *erg11A/cyp51A* expression and heightened azole sensitivity, reinforcing their role as transcriptional activators. These findings underscore the spatially distinct and context-dependent regulation of *erg11A/cyp51A* by TR34 [42]. Crucially, these insights challenge the conventional assumption that TR34-driven overexpression arises simply from increased dosage effects due to repeat amplification. Instead, the distinct roles of proximal and distal elements point to a more sophisticated regulatory interplay. We propose a model where, under normal conditions, a multivalent corepressor complex interacts with SrbA, AtrR, CBC, and HapX to repress *erg11A/cyp51A* transcription. Azole exposure may disrupt this corepressor complex, leading to transcriptional activation. In the TR34 promoter, distal SRE and ATRE elements may escape corepressor-mediated repression, thereby driving the constitutive overexpression of *erg11A/cyp51A* (Figure 3). This refined understanding of TR34’s regulatory dynamics represents a significant advancement in antifungal resistance research. By distinguishing the unique roles of proximal and distal transcription factor binding sites, these findings open the door for novel therapeutic strategies aimed at disrupting TR34-mediated resistance.

#### 4.1.3. Azole Resistance Under Coordinated Regulation of Ergosterol Pathway Remodeling and Drug Efflux

Research indicates that the development of fungal resistance relies on a coordinated mechanism involving the functional remodeling of Cyp51A and the drug efflux mediated by the ABC transporter AbcG1 [40]. In the biosynthesis of ergosterol, Cyp51A functions as a key enzyme, and its activity is affected not only by gene mutations but also by transcriptional regulation. The TR34 promoter repeat mutation enhances gene transcription, compensating for the decrease in protein stability caused by the L98H missense mutation, thereby forming a unique compensatory regulatory pattern. Molecular dynamics simulations reveal that the L98H mutation alters the enzyme’s spatial conformation, weakening the binding affinity of azole drugs without affecting the composition of sterol products, which explains the consistent sterol profiles observed between resistant and sensitive strains [52]. Further studies have demonstrated that the full manifestation of the resistance phenotype requires the synergistic action of Cyp51A and the ABC transporter AbcG1. In TR34/L98H mutant strains, knockout of the *abcG1* gene significantly increases sensitivity to azole drugs. Meanwhile, the transcription factor AtrR establishes a physiological regulatory network between ergosterol synthesis and drug efflux by binding to the promoter regions of both *cyp51A* and *abcG1.* Based on this synergistic mechanism, a novel clinical intervention strategy has been proposed: inhibitors targeting AbcG1 may restore the antifungal activity of azole drugs against TR34/L98H mutant strains to sensitive levels. In addition, the compensatory mechanism of Cyp51A protein stability suggests that attention should be paid to the dynamic interactions within the enzyme complex in resistant mutants, which may affect the efficacy of traditional single-target drugs. Overall, this study systematically elucidates the synergistic interplay between metabolic pathway remodeling and the activation of drug efflux systems in the formation of azole resistance, providing a solid theoretical foundation for the development of multi-target intervention strategies.

#### 4.1.4. Evolutionary Innovations in Ergosterol Transcriptional Networks Across *Candida* Species

The emergence of azole-resistant *Candida* pathogens has revealed striking evolutionary divergence in ergosterol transcriptional regulation, contrasting sharply with the SREBP-dependent systems found in *Aspergillus* and *Schizosaccharomyces*. This regulatory plasticity is central to the development of clinical drug resistance through three key mechanisms, highlighting the distinct strategies that *Candida* species have evolved to cope with azole treatment.

**Figure 4:**
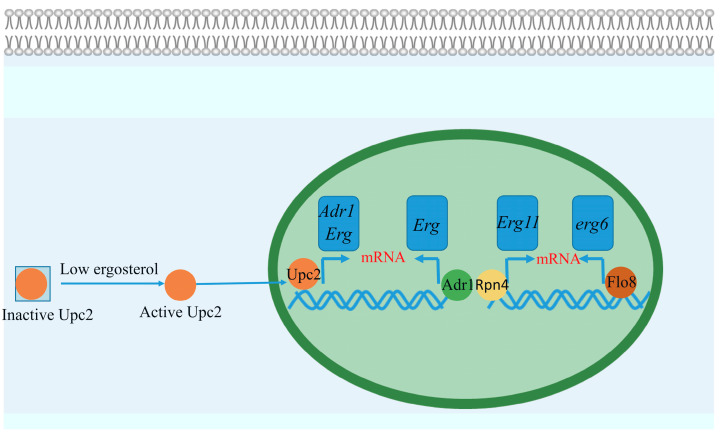
The transcription factors involved in ergosterol biosynthesis in *Candida* Species. The Upc2 transcription factor binds to ergosterol and remains inactive in the cytosol. Depletion of ergosterol changes Upc2 to an active form, which enters the nucleus and initiates the transcription of the genes for ergosterol biosynthesis. In *C. albicans*, activated Upc2 also triggers expression of the Adr1 transcription factor, which further leads to direct expression of ergosterol biosynthesis genes.

Unlike *S. pombe* and *Aspergillus* species, which possess homologs of the sterol regulatory element-binding protein (SREBP), *S. cerevisiae* and *Candida* species lack this homolog and have developed unique mechanisms for regulating *erg11p* expression. In *C. albicans*, the transcription factor Upc2, which functions similarly to SREBP, regulates ergosterol biosynthesis at the transcriptional level (Figure 4). Upc2 directly binds to sterol regulatory elements (SREs) in the promoters of key ergosterol biosynthesis genes, including *erg2p*, *erg7p*, *erg11p*, and *erg25p*, thus controlling their expression [53]. Deletion of Upc2 results in hypersensitivity to azole drugs, illustrating the critical role of Upc2 in azole resistance. Moreover, Upc2 functions as a key regulatory protein for ergosterol biosynthesis in other *Candida* species, such as *C. glabrata* and *C. auris* [54,55]. However, unlike SREBPs, Upc2 does not regulate the basal expression of *erg11p*; its expression remains stable in *Δupc2* strains under non-azole stress conditions, suggesting a context-specific regulation [56].

In addition to Upc2, the transcription factor Adr1 has emerged as another key regulator of ergosterol biosynthesis and azole resistance in *C. albicans* [57]. While Adr1 is known for regulating ethanol, glycerol, and fatty acid metabolism in *S. cerevisiae*, in *C. albicans*, it has evolved a specialized role, with its DNA-binding motif enriched in the promoters of ergosterol biosynthesis genes. Although Upc2 has traditionally been considered the primary regulator of ergosterol biosynthesis, recent studies suggest that Adr1 can independently activate the transcription of these genes [57]. Notably, fluconazole resistance driven by Upc2 activation is heavily dependent on Adr1, whereas a loss of Upc2 has a limited impact on fluconazole resistance in strains with activated Adr1. Furthermore, Upc2 regulates Adr1 expression by binding to a DNA-binding site in the Adr1 promoter, whereas Adr1 does not directly regulate Upc2, indicating that Upc2 serves as a master regulator, controlling ergosterol biosynthesis both directly and indirectly through Adr1. This finding highlights a cooperative and compensatory relationship between these transcription factors (Figure 4), where Adr1 can maintain the expression of ergosterol biosynthesis genes even in the absence of Upc2.

Recent findings have also implicated the transcription factor Rpn4 in azole tolerance in *C. albicans* (Figure 4). A loss of Rpn4 increases sensitivity to azole drugs and decreases transcript levels of several ergosterol biosynthesis genes, including Upc2 [58]. Rpn4 is essential for the constitutive transcription of ergosterol and heme biosynthesis genes, as well as for their full induction in response to fluconazole. Interestingly, the regulatory role of Rpn4 differs between *C. albicans* and *C. glabrata*. In *C. glabrata*, Rpn4 is required for fluconazole-induced gene expression but not for basal expression, as it directly binds to the *erg11p* promoter [59]. In contrast, no Rpn4-binding site has been identified in the *C. albicans erg* gene promoters, suggesting that Rpn4 may regulate ergosterol biosynthesis indirectly, potentially through modulation of Upc2 expression.

The yeast-to-hyphal transition and biofilm formation are critical virulence factors in *C. albicans*, both of which are closely linked to ergosterol biosynthesis. The transcription factor Flo8, which is involved in filamentous growth and biofilm formation, has recently been shown to physically interact with the *erg6p* promoter, directly regulating its transcription (Figure 4). Deletion of Flo8 results in the accumulation of Erg6p substrates, while overexpression of Erg6p partially restores biofilm formation and virulence in Flo8-deficient strains [60]. These findings suggest that Erg6p acts downstream of Flo8, linking sterol biosynthesis to virulence mechanisms in *C. albicans*.

### 4.2. Redox-Driven Regulation of Cyp51/Erg11p: Evolutionary Innovations in Electron Flux Management

The catalytic efficiency of Cyp51/Erg11p, the azole-targeted enzyme central to ergosterol biosynthesis, is regulated by a redox-dependent mechanism that has undergone notable evolutionary diversification across fungal pathogens. Recent research has uncovered two interconnected mechanisms that fine-tune Cyp51/Erg11p activity, extending beyond traditional transcriptional control to include electron flux management and heme-mediated stability control.

#### 4.2.1. Electron Donor Hierarchy and Clinical Resistance

Cyp51/Erg11p is a cytochrome P450 enzyme that requires two electrons for its catalytic activity, supplied by the cytochrome b5 reductase (CB5R) system. This system, which includes cytochrome P450 reductase (CPR), cytochrome b5 (Cyb5), and NADH cytochrome b5 reductase [61], was initially believed to transfer electrons through a two-step process involving NADPH cytochrome P450 reductase as the primary electron donor, with Cyb5 playing a supporting role. However, more recent studies, particularly those investigating azole sensitivity in yeast, have shown that CPR is the key electron donor for Cyp51 activity. Interestingly, overexpression of the Cyb5 gene can increase resistance to ketoconazole in the absence of CPR, suggesting that the CB5R system can provide electrons through multiple pathways simultaneously [62]. This dual-electron delivery mechanism is essential, as a steady supply of electrons is necessary for P450 function. Thus, overexpression of Cyp51A does not always correlate with increased resistance, likely due to limited electron donor availability.

In *A. fumigatus*, two distinct CPRs—CprA and CprB—along with CybE, form a CB5R system that plays a crucial role in regulating Cyp51 enzyme activity [62,63]. Overexpression of Cyp51A increases azole resistance to some extent, but its effectiveness is constrained by electron donor availability. However, simultaneous overexpression of both Cyp51A and CprA significantly enhances azole resistance, establishing CprA as a key enhancer of Cyp51A activity [62,64]. Conversely, overexpression of Cyp51A with either CprB or CybE alone does not show a significant increase in resistance, further emphasizing CprA’s pivotal role. These findings suggest that mutations that enhance CprA activity, such as those in its promoter or regulatory transcription factors, could substantially contribute to azole resistance, particularly in strains with existing Cyp51A overexpression mutations (e.g., TR34/L98H). Therefore, inhibitors of CPRs may represent promising adjunctive therapies, potentially reducing the dosage of azoles required for effective treatment.

Although CybE overexpression does not significantly enhance azole resistance when paired with Cyp51A, its loss leads to the accumulation of the sterol precursor eburicol, lower ergosterol levels, and increased sensitivity to voriconazole (VRZ) [65]. Moreover, CybE loss triggers compensatory upregulation of *cyp51A* and *cprA*, suggesting that CybE plays a regulatory role in maintaining Erg11 function. While CybE may not be crucial for resistance when Cyp51A levels are high, it plays an important role in regulating Erg11p by interacting with other enzymes such as Erg7p and Erg13p to control ergosterol biosynthesis [63]. Notably, CybE, particularly its C-terminal region containing two transmembrane domains, is located in the endoplasmic reticulum, where both the cytochrome b5 reductase system (CybE/Cyb5) and CprA collaborate to supply electrons to Erg11p, ensuring its catalytic activity and maintaining the sterol balance critical for fungal survival.

#### 4.2.2. Heme-Mediated Stability Control

The catalytic activity of Cyp51/Erg11p is closely tied to its stability, which is regulated by proteins such as Dap1 (damage response protein 1). In *S. cerevisiae*, Dap1 stabilizes Erg11p through its interaction with heme, thereby regulating Erg11p protein levels and its activity in ergosterol biosynthesis [66]. In the absence of Dap1, Erg11p substrates accumulate, leading to decreased ergosterol levels and increased sensitivity to Erg11p inhibitors. In *S. pombe*, Dap1 directly binds to Erg11p and other P450 enzymes, whereas a stable interaction is not observed in *S. cerevisiae* [67]. In *A. fumigatus,* members of the Dap protein family—DapA, DapB, and DapC—collectively regulate Cyp51A activity and influence azole susceptibility. DapA stabilizes Erg11p by binding to heme, while DapB and DapC antagonize DapA by depleting heme, thereby reducing Cyp51A activity [68]. This “heme tug-of-war” orchestrated by the Dap family creates a rheostat-like system, where the relative levels of DapA, DapB, and DapC determine Cyp51 activity. Such a regulatory system is absent in *S. cerevisiae*, where Dap1’s influence on Erg11p is indirect and less finely tuned. The Dap1 family’s heme-dependent stabilization of Cyp51/Erg11p represents a novel layer of regulation that contributes to fungal adaptation to azole treatments and highlights the complexity of redox regulation in pathogenic fungi.

## 5. The Ergosterol Biosynthesis Pathway: Innovations in Antifungal Target Discovery and Therapeutic Development

The fungal-specific biosynthesis of ergosterol is a cornerstone of antifungal drug development, providing both target specificity and essentiality while minimizing off-target effects in mammalian hosts. Traditional antifungal therapies primarily target late-stage enzymes in the ergosterol biosynthesis pathway, such as Erg1p, Erg11p (Cyp51), and Erg24p. However, emerging therapeutic strategies are revolutionizing antifungal design by identifying novel targets, introducing dual-action mechanisms, and exploiting natural compounds. These innovations aim to address the critical limitations of current antifungal agents [69,70].

### 5.1. Reimagining Cyp51/Erg11p Inhibition: Beyond Traditional Azoles

Cyp51/Erg11p, the primary target of azole antifungals, has been extensively studied due to its pivotal role in ergosterol biosynthesis. However, clinical use is hindered by challenges such as resistance, toxicity, and pharmacokinetic limitations [70,71,72]. Recent advancements focus on overcoming these issues through structural optimization and the development of novel chemotypes. For example, tetrazole derivatives, such as oteseconazole (FDA-approved in 2022 for recurrent vulvovaginal candidiasis) and VT-1598, exhibit enhanced selectivity by reducing metal affinity and basicity compared to classical imidazole/triazole scaffolds [70,73,74]. While these compounds show promise, their fetal toxicity highlights the need for safer alternatives, which has prompted the exploration of non-azole inhibitors [75,76].

### 5.2. Dual-Target Strategies: Synergistic Mechanisms to Combat Resistance

Histone acetylation modification—which includes both acetylation and deacetylation—is a fundamental aspect of epigenetic regulation. Histone deacetylases (HDACs), enzymes that remove acetyl groups from histones, play a critical role in chromatin remodeling and the regulation of gene expression [77]. In fungi such as *A. fumigatus*, *C. albicans,* and *C. neoformans*, HDACs have been implicated in virulence-related processes and morphological changes [78,79,80]. Consequently, inhibiting fungal HDACs presents a promising strategy for treating fungal infections. Recently, HDAC inhibitors have been proven to be synergized with azole antifungal drugs. For example, the HDAC inhibitor MGCD290 was synergized with fluconazole (FLC) against a variety of clinical fungal isolates [81,82,83].

Clinically, a specific drug combination is important for disease control, whereas drug cocktails have the disadvantages of drug–drug interaction, poor patient compliance, and unpredictable pharmacokinetic (PK) profiles. Therefore, a multi-targeting drug is a feasible solution to retain the synergistic effect and overcome these limitations. A pioneering example is the work of Tianbao Zhu, who developed hybrid inhibitors that target both Cyp51/Erg11 and histone deacetylases (HDACs). These inhibitors exhibit potent antifungal activity while reducing the development of resistance [84,85]. Another exciting development is the observed synergy between terbinafine (a squalene epoxidase/Erg1 inhibitor) and azoles, demonstrating the potential of combining ergosterol biosynthesis inhibitors with complementary mechanisms [69]. Preclinical studies further validate dual-target inhibitors, such as those targeting both Cyp51 and squalene epoxidase, as well as natural compounds like gallic acid and eugenol, which display multi-enzyme inhibitory effects. These compounds provide valuable blueprints for next-generation antifungal therapies [70,86,87].

### 5.3. Unlocking Underexplored Targets: Erg6p and Early-Pathway Enzymes

The species-specific differences in sterol metabolic pathways between fungi and mammals provide valuable insights for antifungal drug development. Ergosterol, a C28 sterol unique to fungi and protozoa, contrasts with cholesterol—the predominant C27 sterol in mammals [15]. While both sterols share a tetracyclic backbone featuring a hydroxyl group at C-3 and a double bond at C-5,6, ergosterol is distinguished by an additional methyl group at C-24 on its side chain [88]. This modification is catalyzed by the fungal-specific enzyme sterol C-24 methyltransferase (Erg6p). Notably, while most enzymes in the fungal ergosterol biosynthesis pathway have mammalian orthologs involved in cholesterol synthesis, Erg6p—together with Erg4p and Erg5p—represents a trio of targets entirely absent in humans [89]. Erg6p displays significant functional diversity among fungal species. In *S. cerevisiae*, it operates at a later stage in the pathway by methylating lanosterol. In contrast, pathogens such as *C. neoformans* exhibit high substrate specificity for lanosterol, positioning Erg6p as an early enzymatic step [90]. The enzyme utilizes an S-adenosylmethionine (SAM)-dependent transmethylation mechanism to add a methyl group at C-24 while concurrently shifting a double bond to form a C-24(28)-methylene structure. These findings confirm that fungi rely on a kinetically favored pathway regulated by 24-sterol methyltransferase (24-SMT) to ensure precise ergosterol synthesis [90,91]. Importantly, inhibiting Erg6p disrupts fungal growth to a degree comparable to clinical antifungals like amphotericin B and itraconazole.

Recent breakthroughs have underscored the pathogenic role of Erg6p and its therapeutic potential. In *C. albicans*, deleting the *erg6p* gene or inhibiting its activity with novel small molecules blocks the yeast-to-hyphae transition, thereby reducing virulence [92]. In *A. fumigatus*, Erg6p localizes to lipid droplets, where it governs ergosterol biosynthesis; this function is essential for in vitro viability and contributes to fungal burden during infection [93]. Collectively, these studies position Erg6p orthologs as central regulators of fungal growth, virulence, and drug susceptibility, reinforcing their promise as fungal therapeutic targets.

Sterol methyltransferase inhibitors have garnered significant attention for their species specificity. The most extensively studied compounds include transition state analogs such as 25-azalanosterol (AZAL) and 24(R,S),25-epiminolanosterol (EIL), along with mechanism-based inhibitors like 26,27-dehydrozymosterol [90,91,94]. Although these agents demonstrate potent inhibition of Erg6p, off-target toxicity has hindered the clinical translation of AZAL and EIL [95]. A pivotal breakthrough came with the discovery of an allosteric small-molecule inhibitor, H55, which maintains high activity against *C. albicans* Erg6p while exhibiting minimal cytotoxicity. H55 monotherapy shows therapeutic efficacy in mouse models of azole-resistant candidiasis. Treatment with H55 increases the accumulation of zymosterol, the substrate of Erg6p. The results of enzyme assays, photoaffinity labeling, molecular simulation, mutagenesis, and cellular thermal shift assays support H55 as an allosteric inhibitor of Erg6p [92]. Collectively, H55, an inhibitor of the fungal-specific enzyme Erg6p, holds potential for treating *C. albicans* infections.

In addition to Erg6p, upstream enzymes such as Erg9p (squalene synthase) and Erg7p (lanosterol synthase) present untapped opportunities for antifungal drug design. Despite challenges in developing selective inhibitors, the complete absence of these enzymes in human sterol metabolism underscores their potential as species-specific targets. Systematic elucidation of the molecular mechanisms of Erg6p and its associated pathways may pave the way for next-generation antifungals with enhanced efficacy and safety profiles, particularly for treating life-threatening infections like invasive aspergillosis.

### 5.4. Direct Ergosterol Targeting and Natural Product Exploration

Azole drugs and polyenes such as amphotericin B remain crucial for treating systemic fungal infections, not only by inhibiting ergosterol biosynthesis but also by directly binding to ergosterol. Meanwhile, natural products are emerging as promising multi-target inhibitors, offering advantages such as antioxidative and anti-biofilm properties that many synthetic antifungal agents lack [96]. As pathogenic fungi continue to develop resistance and existing antifungal drugs are often limited by toxicity and side effects, there is an urgent need for more effective and safer treatment strategies. Due to their low toxicity and multi-target mechanisms, natural products can enhance the efficacy of conventional antifungal drugs, driving research toward combination therapies. These approaches target fungal metabolic pathways—including cell membranes, biofilms, and efflux pump systems—to reduce drug dosages and combat resistance [97]. Studies have shown that Polish propolis combined with fluconazole or voriconazole can synergistically clear biofilms, while carvacrol paired with fluconazole or amphotericin B (AmB) enhances efficacy against *C. albicans* [98]. Additionally, flavonoids such as quercetin and rutin improve the antifungal activity of AmB against *Candida* species and *Cryptococcus* [99]. Furthermore, the combination of propolis with azole drugs, as well as pretreatment with cinnamic acid and propolis, significantly enhances the antifungal effect of AmB, highlighting the strong synergistic potential between natural products and conventional antifungal agents [100,101]. Looking ahead, artificial intelligence and mathematical modeling may further optimize combination therapy by dynamically matching drugs and natural products based on fungal resistance profiles, thereby reducing dosages and shortening treatment durations. However, comprehensive validation through in vitro, animal, and clinical studies remains essential before these strategies can be implemented in clinical practice. At present, natural products should primarily be regarded as enhancers that improve existing treatment regimens rather than as complete replacements for conventional therapies. In conclusion, the ergosterol biosynthesis pathway remains a key target in antifungal research. Advances in Cyp51 inhibitors, dual-target therapies, and natural product derivatives offer potential solutions to challenges such as drug resistance, toxicity, and limited drug spectra. To bridge the gap between preclinical findings and clinical applications, future research must focus on target validation, pharmacokinetic optimization, and translational studies to ensure that these novel therapies achieve their full therapeutic potential.

### 5.5. Nanoparticle-Based Targeted Delivery Systems: Novel Antifungal Strategies Centered on Ergosterol

Currently, the treatment of systemic fungal infections faces numerous challenges, including uneven drug distribution, insufficient efficacy, low targeting, and potential severe side effects, with the increasing issue of drug resistance [102]. Nanotechnology-based antifungal therapies, through the development of nanoparticle (NP)-based targeted and controlled-release drug delivery systems, offer innovative solutions to these problems [103,104]. These therapies not only have the potential to enhance the effectiveness of antifungal treatments but also reduce the negative impacts on the overall health of patients [105]. As a result, this field is attracting significant attention due to its transformative potential in the prevention and treatment of fungal infections [103]. Numerous studies have shown that nanoparticles, due to their small size, multifunctionality, and biocompatibility, can effectively reduce drug side effects, enhance targeting to infection sites, reduce the risk of resistance, and improve the stability and solubility of antifungal drugs, thus improving overall treatment outcomes [106,107,108,109]. Among these, lipid-based nanocarriers are the most widely studied, and many related formulations have entered clinical trials. One significant breakthrough in liposome technology is the commercialization of liposomal amphotericin B [110]. This technology encapsulates the drug within a phospholipid bilayer, preserving the drug’s antifungal activity while significantly reducing its toxicity, allowing for widespread clinical use. Additionally, liposomes have been used to deliver other antifungal drugs, such as fluconazole, where liposomal delivery has demonstrated in vitro activity comparable to or even stronger than that of the free drug [104]. However, further research is still needed to enable broader clinical translation of these nanoparticle-based formulations. Research by Hassanpour et al. has shown that the liposomal formulation of voriconazole has a stronger inhibitory effect on the growth of fluconazole-resistant Candida albicans than voriconazole alone, while also reducing the expression of resistance genes, providing a new option for treating resistant fungal infections [111,112]. Beyond liposomes, other nanoparticle systems such as polymeric nanoparticles and dendrimers have also shown promising potential in antifungal drug delivery. Overall, nanotechnology-based antifungal therapies open new pathways for overcoming the issues of uneven drug distribution, high toxicity, and resistance in traditional antifungal treatments, showing broad prospects for future applications and development.

## 6. Identified Research Gaps and Future Directions

The comprehensive review on ergosterol biosynthesis in pathogenic fungi highlights several critical gaps in current knowledge which present opportunities for future research.

### 6.1. Mechanistic and Functional Gaps in Ergosterol Pathway Components

There are several mechanistic and functional gaps in the ergosterol biosynthesis pathway that require further investigation. The species-specific diversification of enzymes, such as Erg25p and Erg251p in *C. albicans* and *A. fumigatus*, remains poorly understood at the molecular level, necessitating comparative studies to elucidate evolutionary adaptations and their implications for drug targeting. Additionally, the biological function of C-24 ethyl sterol—a rare plant-like sterol found in *A. fumigatus*—is unclear, and its potential roles in environmental adaptation, virulence, or drug resistance warrant further exploration. Furthermore, the interplay between sterol reprogramming, phenotypic adaptation (e.g., hyphal transition, biofilm formation), and environmental stress responses (e.g., hypoxia, immune attacks) is not well characterized. Leveraging systems biology approaches could help map these complex interactions and regulatory networks.

### 6.2. Regulatory Network Complexity

The regulatory complexity of the ergosterol biosynthesis pathway remains largely unresolved, particularly in the areas of epigenetic and post-transcriptional control. The role of non-coding RNAs in modulating ergosterol biosynthesis is underexplored, while the interaction between chromatin modifiers (e.g., histone acetyltransferases) and key transcription factors such as Upc2 and SrbA requires further characterization. Additionally, RNA-binding proteins that influence mRNA stability and translation of ergosterol-related genes remain largely unstudied. Beyond these intracellular regulators, the molecular mechanisms linking environmental cues—such as hypoxia, pH shifts, and nutrient limitation—to ergosterol biosynthesis, potentially mediated by SREBPs or Dap proteins, are still unclear. Unraveling these regulatory networks is essential for a comprehensive understanding of ergosterol homeostasis and its adaptive significance in fungal pathogens.

### 6.3. Therapeutic Strategy Limitations

Therapeutic strategies targeting the ergosterol biosynthesis pathway face several limitations, particularly in the areas of polypharmacology, target validation, and drug delivery. The mechanisms of action for natural products such as curcumin and gallic acid, which act on multiple ergosterol enzymes, remain poorly characterized, necessitating further studies to optimize their antifungal potential. Similarly, synergistic drug combinations, such as azoles with squalene epoxidase inhibitors, require careful optimization to balance efficacy and toxicity. Erg6p inhibitors like H55 have shown promise, but their in vivo validation—assessing toxicity, pharmacokinetics, and potential resistance evolution—remains incomplete. Additionally, while nanoparticle-based antifungal delivery systems, such as liposomal azoles, offer advantages in drug stability and targeting, their clinical translation is hindered by insufficient data on biodistribution, immune interactions, and long-term safety. Addressing these gaps is essential for developing more effective and sustainable antifungal therapies.

## 7. Conclusions

The ergosterol biosynthesis pathway remains a critical and dynamic frontier in antifungal drug discovery, offering diverse opportunities to combat resistance, enhance therapeutic efficacy, and disrupt fungal pathogenicity. This review highlights several innovative strategies aimed at exploiting this pathway, focusing on three transformative directions.

### 7.1. Reinvigorating Azoles Through Synergistic Mechanisms

Azole resistance continues to pose a significant challenge to global antifungal efficacy, but emerging combination therapies are reshaping the potential of azoles. Co-administering ergosterol biosynthesis inhibitors—such as squalene epoxidase (Erg1p) or C-24 sterol methyltransferase (Erg6p) blockers—alongside azoles can overcome resistance by disrupting multiple pathways, thereby reducing the evolutionary pressure that drives resistance. For instance, terbinafine–azole combinations have shown clinical success against azole-resistant *C. albicans*, while preclinical dual-target inhibitors, such as Cyp51/HDAC hybrids, demonstrate enhanced fungicidal activity and reduced toxicity. These strategies not only resensitize resistant strains but also lower the required drug doses, reducing the hepatotoxicity risks associated with prolonged high-dose monotherapy.

### 7.2. Targeting Fungal Virulence and Host Adaptation

In addition to inhibiting fungal growth, disrupting ergosterol biosynthesis can undermine key virulence mechanisms crucial for host colonization. Depleting ergosterol destabilizes fungal membrane integrity, impairing stress response pathways, nutrient uptake, and biofilm formation. Targeting enzymes such as Erg6p or Erg25p can attenuate *C. albicans* hyphal transition and reduce tissue invasion, effectively reverting the pathogen to an avirulent state. This “virulence-centric” approach aligns with evolving antifungal strategies that prioritize pathogen debilitation over complete eradication, potentially reducing host inflammation and recurrence rates.

### 7.3. Harnessing Regulatory Networks for Precision Targeting

Emerging insights into the regulation of the ergosterol biosynthesis pathway have uncovered novel targets for synergistic intervention. Master regulators such as SREBPs (sterol regulatory element-binding proteins), Dap family proteins, and CprA play central roles in maintaining ergosterol homeostasis and mediating antifungal resistance. Inhibiting these regulators—through small-molecule inhibitors or epigenetic modulators—could destabilize fungal adaptive responses. Recently identified regulators, including Rpn4, Flo8, and SltA, offer significant potential for species-specific therapies. For example, targeting AtrR in *A. fumigatus* could block the upregulation of efflux pumps, a critical mechanism of azole resistance.

### 7.4. Integrating Omics and Computational Tools for Next-Generation Inhibitors

Advances in multi-omics (genomics, proteomics, lipidomics) and AI-driven drug design are accelerating the discovery of fungal-specific inhibitors. Natural products like gallic acid and eugenol, which inhibit both Cyp51 and squalene epoxidase, exemplify the potential of phytochemicals as multi-target scaffolds. Additionally, structure-guided optimization of anti-pyridine derivatives, such as H55, against Erg6p demonstrates the power of computational modeling in refining selectivity and potency. Future research should prioritize compounds with dual-target capabilities, low host toxicity, and broad-spectrum activity, particularly against emerging multidrug-resistant species like *C. auris* and *Fusarium.* These strategies position the ergosterol biosynthesis pathway as a dynamic target for the development of next-generation antifungal therapies, holding promise for overcoming resistance, improving efficacy, and expanding therapeutic options in the fight against fungal infections.

## Data Availability

Data sharing is not applicable to this article as no new data were created or analyzed in this study.
